# Sugar Signaling and Post-transcriptional Regulation in Plants: An Overlooked or an Emerging Topic?

**DOI:** 10.3389/fpls.2020.578096

**Published:** 2020-11-05

**Authors:** Ming Wang, Lili Zang, Fuchao Jiao, Maria-Dolores Perez-Garcia, Laurent Ogé, Latifa Hamama, José Le Gourrierec, Soulaiman Sakr, Jingtang Chen

**Affiliations:** ^1^College of Agronomy, Qingdao Agricultural University, Qingdao, China; ^2^IRHS-UMR1345, INRAE, Institut Agro, SFR 4207 QuaSaV, Université d’Angers, Beaucouzé, France

**Keywords:** sugar, RNA-binding protein, post-transcriptional regulation, microRNA, mRNA stability

## Abstract

Plants are autotrophic organisms that self-produce sugars through photosynthesis. These sugars serve as an energy source, carbon skeletons, and signaling entities throughout plants’ life. Post-transcriptional regulation of gene expression plays an important role in various sugar-related processes. In cells, it is regulated by many factors, such as RNA-binding proteins (RBPs), microRNAs, the spliceosome, etc. To date, most of the investigations into sugar-related gene expression have been focused on the transcriptional level in plants, while only a few studies have been conducted on post-transcriptional mechanisms. The present review provides an overview of the relationships between sugar and post-transcriptional regulation in plants. It addresses the relationships between sugar signaling and RBPs, microRNAs, and mRNA stability. These new items insights will help to reach a comprehensive understanding of the diversity of sugar signaling regulatory networks, and open onto new investigations into the relevance of these regulations for plant growth and development.

## Introduction

As living organisms, plants need various compounds to meet the requirements of their global metabolism and to finely adapt to different external stimuli. In this process, one of the most essential compounds is sugar, which has both trophic and signaling activities during plant development – a high-energy-demanding and well-controlled process. Plants synthesize sugar from carbon dioxide and water through photosynthesis and finely tune their sugar status to avoid sugar starvation (Stitt and Zeeman, 2012; Kanwar and Jha, 2019; Signorelli et al., 2019). They have evolved a sophisticated machinery to sense different forms of sugars, including hexoses, sucrose, and various sugar phosphates (e.g., trehalose-6-phosphate), and elicit the appropriate responses. Some responses are sugar-type-specific (Granot et al., 2014; Figueroa and Lunn, 2016; Li and Sheen, 2016; Janse van Rensburg and Van den Ende, 2018; Wingler, 2018). As a signaling entity, sugar can influence a diversity of physiological processes of the plant life cycle and operates on transcriptional, post-transcriptional, translational, and post-translational regulation. Most of the currently available knowledge is focused on sugar-dependent transcriptional regulation (Lastdrager et al., 2014; Li and Sheen, 2016; Sakr et al., 2018; Wingler, 2018; Jiao et al., 2019; Rodriguez et al., 2019; Sami et al., 2019). Post-transcriptional regulation of gene expression is a pivotal mechanism whereby plants rapidly reprogram their transcriptome and proteome in response to endogenous and environmental cues and involves many factors such as proteins [RNA-binding proteins (RBPs)], microRNAs (miRNAs), and the spliceosome (Guerra et al., 2015; Romanowski and Yanovsky, 2015; Zhang, 2015; Kawa and Testerink, 2017; Samad et al., 2017; Marondedze et al., 2019; Rigo et al., 2019). RBPs are mainly cytosolic and nuclear proteins that govern the processing, cellular localization, and decay of cellular RNA. They contain RNA recognition motifs (RRMs) that allow their binding to a specific sequence in the target transcripts (Chou et al., 2017; Tian et al., 2018a; Lu et al., 2019; Mahalingam and Walling, 2020). miRNAs are small non-coding RNA molecules that function in RNA silencing *via* base-pairing with complementary sequences within mRNA molecules (Bartel, 2004, 2018), leading to mRNA cleavage; they shorten the poly(A) tail of mRNAs, or influence mRNA translation, altogether downregulating gene expression through the target transcript (Zhang, 2015; Meyers and Axtell, 2019). Alternative splicing – also termed alternative RNA splicing – is mediated by the spliceosome, a complex and large molecular machinery mainly located within the nucleus of eukaryotic cells (Wilkinson et al., 2020). Splicing can also have other functions, like the generation of premature stop codons that recruit the nonsense-mediated decay (NMD) machinery (Kesarwani et al., 2019; Wilkinson et al., 2020). Consequently, the proteins translated from alternatively spliced mRNAs are expected to have different amino acid sequences, different protein structures, and even different biological functions.

Sugar signaling-dependent regulation represents an intricate regulatory network that relies on highly diverse mechanisms that coordinate the appropriate use of available energy and sugar to sustain plant development and growth under the ever-changing environment. Sugar-dependent post-transcriptional regulation could be one important mechanistic aspect of this network. Current knowledge in this topic is still fragmented and makes it very difficult to draw a comprehensive scheme and bring out new research questions. The present review addresses the relationship between sugar and post-transcriptional gene regulation in plants and provides first insights into the role of various important mechanisms of post-transcriptional regulation, i.e., RBPs, miRNAs, and mRNA decay/stability in sugar-related pathways. It underlines the physiological relevance of such regulation mechanisms in different biological contexts and raises questions for upcoming studies.

## Sugar and RNA-binding Proteins

**Figure 1:**
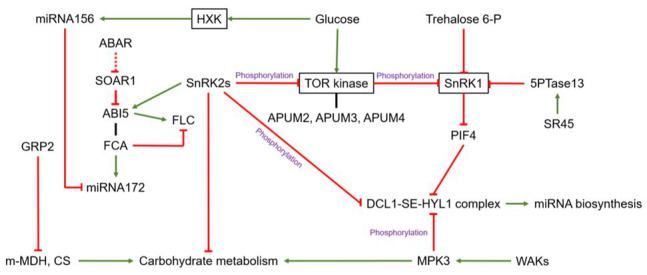
The relationship between sugar and RNA-binding proteins (RBPs), the crosstalk between miRNA and sugar related RNA binding proteins, and alternative splicing in *Arabidopsis*. The green arrow means stimulation or positive effect, the red line means inhibitory effect, and the black line means protein interaction. GRP2, GLYCINE RICH PROTEIN 2; m-MDH, mitochondrial malate dehydrogenase; CS, citrate synthase; FCA, FLOWERING CONTROL LOCUS A; FLC, FLOWERING LOCUS C; ABI5, ABA-insensitive 5; SOAR1, SUPPRESSOR OF THE ABAR OVEREXPRESSOR 1; DRM2, DORMANCY-ASSOCIATED GENE2; SEN1, SENESCENCE-ASSOCIATED GENE1; ASN1, GLUTAMINE-DEPENDENT ASPARAGINE SYNTHETASE1; 5PTase13, 5-phosphatase 13; SR45, serine/arginine-rich 45; PIF4, phytochrome-interacting factor 4; MPK3, MITOGEN-ACTIVATED PROTEIN KINASE 3; WAKs, WALL-ASSOCIATED KINASES.

RNA-binding proteins control nearly all aspects of eukaryotic post-transcriptional gene regulation and consequently determine the fate and expression of the plant transcriptome. Hundreds of RBPs have been identified in *Arabidopsis*. Most of them are plant specific, and could carry out specific functions in plant physiology (Lorković, 2009, Reichel et al., 2016). They share one or more canonical RNA-binding domains including the RRM, the K-homology (KH) domain, the Pumilio/FBF (PUF) domain, the RRM and KH domains, DEAD/DEAH boxes, zinc-finger structures, the Piwi/Argonaute/Zwille (PAZ) domain, double-stranded RNA-binding domains (DS-RBD), pentatricopeptide-repeat (PPR) domains, etc. (Silverman et al., 2013; Lee and Kang, 2016; Wang et al., 2018a). The link between glucose signaling and RBP-mediated post-transcriptional regulation has been explored in the model plant *Arabidopsis thaliana*. Transgenic *Arabidopsis* plants overexpressing *atRZ-1a*, which encodes a zinc-finger-containing glycine-rich RNA-binding protein (GRP), exhibited delayed germination and seedling growth under abiotic stresses (dehydration or salt stress), and hypersensitivity to glucose and ABA, relatively to the wild type (Kim and Kang, 2006; Kim et al., 2007b). Yet, the molecular function of *atRZ1* in glucose-dependent post-transcriptional regulation of seedling establishment is still unknown. The RBP FLOWERING CONTROL LOCUS A (FCA) contains two RRM domains and one WW domain (Jang et al., 2009; Table 1) and operates as an inhibitor of FLOWERING LOCUS C (FLC; Macknight et al., 1997, Liu et al., 2007), one of the repressor integrators, tightly controls flowering signals (Michaels and Amasino, 1999). FLC is positively and transcriptionally regulated by the ABI5 transcription factor (ABA-insensitive 5, Figure 1), which is involved in ABA-mediated floral transition (Wang et al., 2013) and in integrating glucose and ABA-signaling during early seedling development of *Arabidopsis* (Arroyo et al., 2003; Dekkers et al., 2008). AtSOAR1 (SUPPRESSOR OF THE ABAR OVEREXPRESSOR 1) encodes a dual-localized (cytoplasm-nucleus) pentatricopeptide repeat (PPR) protein repeat (Mei et al., 2014; Jiang et al., 2015). By binding to the mRNA of *ABI5*, it represses ABI5 translation in the regulatory cascade downstream of a putative ABA receptor (ABAR; Bi et al., 2019). At the transcriptional level, the transcription factor RAV1, a member of the RAV (Related to ABI3/VP1) subfamily (Riechmann et al., 2000; Feng et al., 2005), binds directly to the *ABI5* promoter and represses its expression, which is alleviated when RAV1 is phosphorylated by ABA-activated sucrose-non-fermenting-1-related protein kinase-2s (SnRK2s; Feng et al., 2014). SnRK2s is a central node that integrates plant growth and development with ABA signaling and environmental stresses (Zheng et al., 2010; Zhang et al., 2011; Shinozawa et al., 2019), partially through dissociation and inhibition of the target of rapamycin (TOR) kinase complex (Wang et al., 2018b; Figure 1). TOR kinase is, itself, an evolutionary conserved master regulator that integrates nutrients, hormones, and energy to promote cell proliferation (Dobrenel et al., 2016; Rosenberger and Chen, 2018; Shi et al., 2018). Interestingly, TOR kinase can directly phosphorylate APUM2, APUM3, and APUM4, three PUF proteins in *Arabidopsis* (Figure 1), providing a direct link between the nutrient status and the activity of RBPs (Table 1; Van Leene et al., 2019). Although their genuine activity is still unclear, APUM-1 to APUM-6 might act as regulators of stem cell maintenance in the shoot meristem (Francischini and Quaggio, 2009), where TOR kinase signaling is required for integrating sugar, hormone, and environmental signals (Li et al., 2017). The expression of *Rosa hybrida PUF4* (*RhPUF4*, an ortholog of *APUM2*) is upregulated by sucrose before the onset of bud outgrowth and may contribute to the promotion of sugar-mediated shoot branching by binding to the 3'UTR of *RhBRC1* (Wang et al., 2019a), a main repressor hub of shoot branching (Wang et al., 2019b). Furthermore, pharmacological disruption of the oxidative pentose phosphate pathway (OPPP) alters sucrose-related *RhPUF4* upregulation and *RhBRC1* downregulation, suggesting a major role of the OPPP in this process (Wang et al., 2019a). The fact that TOR kinase could mediate the upregulation of glucose-6-phosphate dehydrogenase (G6PD, one of key enzymes of the OPPP) and the activity of TOR kinase is probably under the positive regulation of NADPH, a product of the OPPP (Corradetti and Guan, 2006; Liu and Bassham, 2010), it would be noteworthy to investigate the crosstalk between these two pathways in this post-transcriptional process. In addition, although these findings suggest a plausible role of TOR kinase and the OPPP in sugar-mediated RBP-dependent post-transcriptional regulation, questions about whether additional sugar signaling could contribute to this regulation and the nature of the underlying molecular mechanisms are still open.

**Table 1:** The type and function of sugar related RBPs.

Gene	RNA-binding protein type	Function
AtRZ-1a	Zinc finger-containing glycine-rich RNA-binding protein	Involve in freezing tolerance and cold stress
GLYCINE RICH PROTEIN 2	RRM protein	Response to cold, osmotic stress, water deprivation, and seed germination
FLOWERING CONTROL LOCUS A	RRM protein and contain WW domain	Involved in the promotion of the transition of the vegetative meristem to reproductive development
HYPONASTIC LEAVES 1	No reports	Flowering, leaf, and root development
APUM2, APUM3, and APUM4	Pumilio/FBF protein	No reports
ALDH7B4	No reports	Aldehyde dehydrogenase
RAFFINOSE SYNTHASE 6	No reports	Biosynthesis of the raffinose; response to cold, karrikin, and oxidative stress
SOAR1	PPR protein	ABA responses, probably located upstream of an ABA-responsive transcription factor ABI5
RhPUF4	Pumilio/FBF protein	Involved in bud outgrowth probably

The function of *Arabidopsis* protein is from the annotation of TAIR database (www.arabidopsis.org; Swarbreck et al., 2007; Berardini et al., 2015).

RNA-binding-proteins can also directly regulate the sugar metabolism by triggering sugar metabolism-related enzymes. GLYCINE RICH PROTEIN 2 (GRP2), a cold-induced zinc-finger-containing GRP (Fusaro et al., 2007), negatively affects germination, in interaction with ABA and glucose (Kim et al., 2007a). GRP2 can interact with mitochondrial malate dehydrogenase (m-MDH) and citrate synthase (CS), two enzymes of the tricarboxylic acid cycle (TCA), probably leading to an adjustment of the sugar metabolism (Kim et al., 2007a; Figure 1). The response of GRP2 to other environmental cues and to endogenous factors, including sugars, deserves to be investigated to evaluate the physiological relevance of this regulation. Interestingly, sugar metabolism-related enzymes could both include RBPs and display metabolic activities. Based on an interactome capture technique in *Arabidopsis* cell cultures and leaves, Marondedze et al. (2016) identified 18 RBPs involved in glycolysis, and 15 involved in the glyoxylate and dicarboxylate metabolism, while their respective target mRNAs are still unknown. A similar plausible dual function was also reported for RAFFINOSE SYNTHASE 6 (RS6), a metabolic enzyme, involved in the biosynthesis of the raffinose family oligosaccharides and ALDEHYDE DEHYDROGENASE 7B4 (ALDH7B4; Fujiki et al., 2000; Hou and Bartels, 2015; Reichel et al., 2016; Gilmonreal et al., 2017; Marondedze et al., 2019) in different biological contexts (Table 1). ALDH7B4 protein accumulates abundantly in response to abiotic stress and function as aldehyde-detoxifiying enzymes and ROS scavengers enzymes (Kirch et al., 2005; Zhao et al., 2017). Zhao et al. (2018) demonstrated that *ALDH7B4* is a direct target of the NO APICAL MERISTEM/ARABIDOPSIS TRANSCRIPTION ACTIVATION FACTOR/CUP-SHAPED COTYLEDON (NAC) transcription factor ARABIDOPSIS TRANSCRIPTION ACTIVATION FACTOR1 (ATAF1) that integrate carbon starvation responses and trehalose metabolism (Garapati et al., 2015). These findings open onto new investigations on the functional role of their respective target mRNAs and their role in sugar signaling-dependent post-transcriptional regulation.

## Sugars and microRNAs

**Figure 2:**
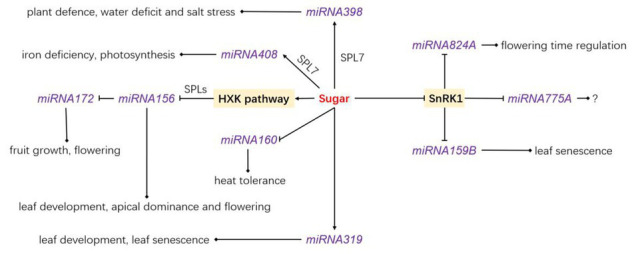
The relationship between sugar and reported *miRNA*, and the function of the related *miRNA*. Sugar stimulates the transcription of *miRNA398*, *miRNA408*, and *miRNA319* but inhibits that of *miRNA160* and *miRNA156*. SnRK1 inhibits the transcription of *miRNA824A*, *miRNA775A*, and *miRNA159B*. HKX1, *hexokinase 1*; SPL, *SQUAMOSA-PROMOTER BINDING PROTEIN-LIKE*.

miRNAs are small non-coding RNA molecules that participate in RNA silencing and post-transcriptional regulation of gene expression (Song et al., 2019). *miRNA* genes are transcribed by RNA polymerase II in the nucleus. This generates long primary transcripts of miRNA (primary miRNAs, pri-miRNA in short), which are converted into a precursor miRNA (pre-miRNA) by endonuclease DICER-like1 (DCL1). After a complex processing involving the C2H2-zinc finger protein SERRATE (SE), DCL1 and the double-stranded RBP HYPONASTIC LEAVES1 (HYL1), the miRNA is loaded onto ARGONAUTE1 (AGO1) to integrate the RNA-induced silencing complex (RISC; Voinnet, 2009; Rogers and Chen, 2013). Several studies support a direct link between sugar signaling and miRNAs in a variety of physiological processes in plants. Duarte et al. (2013) shown that *Arabidopsis* mutants disrupted in miRNA biosynthesis (*hyl1-2* and *dcl1-11*) and miRNA activity (*ago1-25*) exhibited a glucose-hyposensitive phenotype at the early seedling stage, and the expression of several miRNA target genes was deregulated, mainly *via* hexokinase-independent pathway. miRNA156 is one of the best characterized miRNAs in terms of sugar-dependent regulation. It is conserved in land plants and contributes to diverse physiological processes such as leaf development, heat stress memory, developmental transition, apical dominance, and flowering (Kim et al., 2012; Bhogale et al., 2014; Yu et al., 2015; Zhang, 2015; Gao et al., 2018; Kumar et al., 2020). The biological function of miRNA156 implies the repression of SQUAMOSA-PROMOTER BINDING PROTEIN-LIKEs (SPLs; Wang et al., 2009; Wahl et al., 2013; Xu et al., 2016; Wei et al., 2017; Zheng et al., 2019a,b; Hu et al., 2020, Jiao et al., 2020; Ponnu et al., 2020). A direct link between sugar and miRNA156 abundance is based on the ability of exogenous glucose or sucrose supply to cause the levels of mature miRNA156 to drop and thereby accelerate the vegetative-reproductive phase transition, along with the juvenile-to-adult phase transition. Conversely, defoliation and a reduced photosynthetic rate delay plant developmental transitions (Yang et al., 2013; Yu et al., 2013). The glucose-induced repression of miRNA156 is dependent on the hexokinase 1-signaling pathway (Yang et al., 2013), while trehalose-6-phosphaste regulates developmental transition through a distinct mechanism (Wahl et al., 2013; Ponnu et al., 2020). miRNA156 also targets a variety of mRNAs that encode regulatory proteins involved in various physiological processes in plants (Wang and Wang, 2015). Like miRNA156, miRNA399 was determined to be sucrose-responsive through a microRNA array assay and high levels of sucrose inhibited the accumulation of microRNA399 family under phosphate starvation conditions in *Arabidopsis* (Tian et al., 2018b). miRNA398, that is associated with the adaptive plant response to biotic, abiotic, and nutrient stresses and could be involved in sugar-signaling pathway (Sunkar et al., 2006; Dugas and Bartel, 2008; Jia et al., 2009; Feng et al., 2015). *miRNA398* accumulation is repressed by carbon depletion (Pant et al., 2009), while sucrose supply induces its accumulation through the SPL7 transcription factor that directly recognizes the GTAC boxes located in the *miRNA398* promoter (Yamasaki et al., 2009). In line with this, *spl7* knockdown mutants consistently accumulate lower levels of miRNA398 under normal conditions (Ren and Tang, 2012). Targets of miR398a include two ROS-scavenging enzymes (COPPER/ZINC SUPEROXIDE DISMUTASE, CSD1 and CSD2) necessary for detoxification of stress-dependent reactive oxygen species stimulation (Farooq et al., 2019) and this sugar-mediated regulation of *miRNA398* would be an appropriate response to nutrient stress. The *miRNA398* binding site of *CSD1* can be eliminated by alternative splicing in peanut and *Arabidopsis*, resulting in different tolerance levels to abiotic stress (Park and Grabau, 2017), indicating how alternative splicing processes influence plant response through interactions with miRNAs. Microarray analyses have also shown responsiveness to sucrose from other mature miRNAs in *Arabidopsis* (Figure 2), including miRNA408 (involved in the response to iron deficiency and in photosynthesis; Pan et al., 2018; Carrió-Seguí et al., 2019), miRNA319 (involved in leaf development; Koyama et al., 2017), and miRNA160 (involved in heat tolerance; Lin et al., 2018). The levels of miRNA319 and miRNA408 are enhanced by sucrose supply, while the levels of miRNA160 are reduced. Moreover, the induction of miRNA408 by sucrose is associated once again with SPL7 (Ren and Tang, 2012), which may play a prominent role in sugar-mediated regulation of miRNA biosynthesis (Figure 2). In sweet sorghum (*Sorghum bicolor*), the expression levels of nine known mature miRNAs and 12 novel mature miRNAs have been found influenced by sugar abundance in the stem (Yu et al., 2015). Although the targets of these mature miRNAs exhibit functions related to shoot apical meristem specification, polar specification of the adaxial/abaxial axis, bilateral symmetry determination, and transcriptional regulation (Yu et al., 2015), the genuine participation of sugar sensing and signaling in this regulatory network remains to be elucidated.

A link between sucrose transporters (SUTs, H^+^/Suc symporters) and miRNAs exists in plants. SUTs are key players in sucrose phloem loading and sugar allocation within plants (Braun et al., 2014; Milne et al., 2017; Doidy et al., 2019). The half-life of SUT mRNAs ranges between 60 and 130 min and is tightly related to miRNA regulation (He et al., 2008; Liesche et al., 2011). Interestingly, the relationship between miRNA biosynthesis and the cellular energy status is also supported by the fact that the transient overexpression of the energy-sensing SnRK1 in protoplasts leads to the repression of a variety of miRNAs (Confraria et al., 2013). These include miRNA159B (involved in leaf senescence, Huo et al., 2015), miRNA161 (induces the expression of PPR genes, Cai et al., 2018), miRNA775A (no function reported to date), and miRNA824A (involved in flowering time regulation, Hu et al., 2014) and might be involved in SnRK1-dependent energy signaling. However, the molecular regulatory network involved in this SnRK1-dependent miRNA biosynthesis remains an open question.

## Crosstalk Between Sugar-Related RNA-binding Proteins and microRNAs

Post-translational modifications have been reported as a key regulator of the miRNA-biogenesis machinery. In *Arabidopsis*, HYL1 activity is controlled by its phosphorylation state through complex mechanisms. The Protein Phosphatase 4 (PP4)/Suppressor of MEK 1 (SMEK1) complex and C-TERMINAL DOMAIN PHOSPHATASE-LIKE 1 and 2 (CPL1 and CPL2) dephosphorylate and activate HYL1, while Mitogen-activated Protein Kinase (MPK) phosphorylates and inactivates it (Manavella et al., 2012; Su et al., 2017; Meng et al., 2018, Wang et al., 2019c). MPK might bridge a gap between miRNA biosynthesis and sugar signaling, based on its transducing role the WALL-ASSOCIATED KINASE (WAK)-dependent regulation of vacuolar invertase, a driver of cell expansion and growth and a player of sugar signaling (Kohorn et al., 2006, 2009; Figure 1). SnRK2s can also affect the phosphorylation status of HYL and SE (Yan et al., 2017), and it will be very interesting to explore the sensitivity of these two proteins to the kinase activity of SnRK1 and, thereby, its relevance in SnRK1-dependent miRNA biosynthesis regulation (Confraria et al., 2013). Beyond this, the basic helix-loop-helix (bHLH) transcription factor phytochrome-interacting factor 4 (PIF4) interacts directly with DCL1 and HYL1 to promote their destabilization and regulate the processing of primary miRNAs during the dark-to-red-light transition (Sun et al., 2018). PIF4 is also controlled through the trehalose-6-phosphate pathway and SnRK1 to modulate *Arabidopsis* hypocotyl elongation in response to high temperature (Delatte et al., 2011; Hwang et al., 2019), so it might be seen as a hub integrating sugar signaling and environmental cues to modulate the regulation of miRNA biogenesis through the DCL1-SE-HYL complex.

Many previous studies indicate that various RBPs participate in miRNA homeostasis. For instance, the WD-40 protein PLEIOTROPIC REGULATORY LOCUS1 (PRL1) is required for miRNAs and small siRNAs to accumulate, by stabilizing pri-miRNAs through its RNA-binding activity and enhancing DCL1 activity (Zhang et al., 2014). Beyond this function, PRL1 acts as a global regulator of sugar, stress, and hormone responses, partly through SnRK1 repression (Flores-Pérez et al., 2010). However, additional investigations are required to elucidate the molecular connections between these different PRL1-dependent regulatory mechanisms. miRNA172 is a downstream component of the regulatory cascade involved in the regulation of flowering time by sugar-dependent miRNA156 repression (Wu et al., 2009; Martin et al., 2010), in which miRNA172 acts as an inducer of FLOWERING LOCUS T (FT) expression. FCA, an RBP, binds to the flanking sequences of the stem-loop within primary miRNA172 transcripts (pri-mRNA172) *via* the RRM, and promotes its accumulation in response to ambient temperature (Figure 1). FCA also binds to the primary transcripts of other temperature-responsive miRNAs, such as miRNA398 and miRNA399 (Jung et al., 2012). The RBP TOUGH (TGH) contributes to the pri-miRNA-HYL1 interaction (Ren et al., 2012), while MOS2 (MODIFIER OF SNC1, 2) is involved in pri-miRNA processing (Wu et al., 2013). Many other examples exist, including EMBRYO DEFECTIVE 2793 (EMB2793, THO2), MOS4-ASSOCIATED COMPLEX 7 (MAC7), and REGULATOR OF CBF GENE EXPRESSION 3 (RCF3) which participate in the regulation of miRNA biogenesis by interacting with HYL1 (Francisco-Mangilet et al., 2015; Karlsson et al., 2015; Jia et al., 2017). However, whether other core components of miRNA processing are dependent on RBPs and the way sugar signaling could contribute to this regulatory network still remains unclear.

## Sugar and mRNA decay/stability

In plants, mRNA decay/stability is an important control point in the regulation of gene expression and can discard potentially deleterious errors in mRNA synthesis (Nagarajan et al., 2019). The mRNA decay/stability of many sugar-metabolism-related enzymes is controlled through post-transcriptional regulation. This holds true for the maize cell wall invertase gene (*Incw1*) that displays two transcripts – *Incw1-S* (small) and *Incw1-L* (large) – according to the respective lengths of its 3'untranslated regions (UTR; Cheng et al., 1999). Since sucrose and D-glucose appear to be associated with the increased steady-state abundance of *Incw1-S* mRNA and cell wall invertase activity, these authors suggested that the 3'UTR of the *Incw1* gene was a regulatory sensor of carbon starvation and acted as a link between translation activity and the sink metabolism in plants. The 3'UTRs of *OsVIN1* and *AtvacINV2*, encoding vacuolar invertases in rice and *Arabidopsis*, respectively, are involved in this process. Downstream regulatory elements or a motif that participates in the rapid degradation of mRNAs, e.g., small auxin-up RNAs (SAUR; Feldbrügge et al., 2002; van Mourik et al., 2017), may be involved too (Huang et al., 2007). The expression of α-amylase, an endo-amylolytic enzyme that catalyzes starch degradation in plants, is induced by sucrose starvation and suppressed by sucrose availability in rice. Sugar repression of α-amylase 3 (*αAMY3*) expression in rice suspension cells involves controlling both its transcription rate and mRNA stability (Sheu et al., 1994; Chan and Yu, 1998). An analysis of reporter mRNA half-lives indicated that two subdomains of the *αAMY3* 3'UTR contained the UAUAUAUGUA motif required for the sugar-dependent destabilization of *αAMY3* mRNA (Sheu et al., 1994; Chan and Yu, 1998). The same motif might also be involved in sugar-mediated post-transcriptional downregulation of *RhBRC1* in *R. hybrida* (Wang et al., 2019a), and could be conserved in angiosperms. Such sugar-dependent regulation of mRNA stability is required for the rapid adjustment of gene expression in response to the sugar status of the cell. In *Arabidopsis* cell cultures, the stability of 224 mRNAs was repressed by sucrose limitation, concomitantly with a drop in the cell metabolic activity (Nicolai et al., 2006). The mRNA half-lives of actin (*ACT*), alcohol dehydrogenase 2 (*ADH2*), glyceraldehyde 3-phosphate dehydrogenase (G3PD), and sucrose synthase P-2 (*SSP2*) were consistently 1.6‐ to 2.6-fold longer in sucrose-supplied rice cells (Ho et al., 2001). In line with this, the mRNA stability of the bZIP63 transcription factor, an important mediator of the adaptive response induced by SnRK1 during energy or sugar depletion (Baena-González et al., 2007) decreased following exogenous glucose supply in *Arabidopsis* seedlings (Matiolli et al., 2011). The involvement of bZIP63 as a hub integrating the sugar and energy statuses and mRNA stability deserves to be addressed. *Low-β-amylase1* (*lba1*) is a missense mutation of UP-FRAMESHIFT 1 (UPF1) RNA helicase, involved in nonsense-mediated mRNA decay (NMD). Its *Arabidopsis* mutant exhibited lower sugar induction of the *AtβAmy* transcript, which was restored by complementation of the *lba1* mutation with wild type UPF1, further supporting the link between sugar signaling and the fate of the mRNA (Yoine et al., 2006). All these findings clearly indicate a relationship between the sugar status and mRNA stability in a variety of biological contexts, opening the avenue for deciphering the sugar sensing and signaling mechanisms. In line with that, mRNA stability might also be important for the diurnal regulation of mRNA levels of sucrose transporters and in turn in sugar allocation at the whole plant level. For example, a sucrose transporter (SUT1) displayed a quick turnover rate in leaves of tomato (*Lycopersicon esculentum*), potato (*Solanum tuberosum*), and tobacco (*Nicotiana tabacum*; Kühn et al., 1997; Kühn and Grof, 2010). The mRNA levels of *StSUT2* and *StSUT4* may be regulated by putative RBPs (He et al., 2008). Two AUUUA motifs exist in the 3'UTR or CDS region of *StSUT2* and *StSUT4* mRNA (He et al., 2008); they have been characterized as the binding sites of proteins involved in mediating mRNA degradation (Chen and Shyu, 1995; He et al., 2008; Liesche et al., 2011). However, the nature of these proteins is still unknown.

## Sugar and Alternative Splicing

Alternative splicing is a finely regulated process that takes place during gene expression and leads to a single gene coding for multiple proteins. Serine/arginine-rich 45 (SR45) is a serine/arginine-rich splicing factor that participates in 5' and 3' splicing site selection of introns and can bridge the 5' and 3' components of the spliceosome. The SR45 splicing factor regulates glucose signaling during early seedling development in *Arabidopsis* (Carvalho et al., 2010), more likely through the modulation of SnRK1-stability (Carvalho et al., 2016). The *sr45-1* knockout mutant indeed displays a high level of the energy-limitation-sensing SnRK1 protein under glucose supply, which is in agreement with the upregulation of SnRK1-activated genes such as *SENESCENCE-ASSOCIATED GENE1* (*SEN1*), *GLUTAMINE-DEPENDENT ASPARAGINE SYNTHETASE1* (*ASN1*), and *DORMANCY-ASSOCIATED GENE2* (*DRM2*). Moreover, the glucose hypersensitivity of the *sr45-1* mutant is alleviated when SnRK1 is disrupted (Figure 1). SR45 controls the alternative splicing of *5-phosphatase 13* (*5PTase13*) in *Arabidopsis*, which encodes an inositol polyphosphate 5-phosphatase involved in regulating SnRK1 stability negatively *in vitro* (Carvalho et al., 2016; Figure 1). This link between sugar signaling and RNA splicing has also been reported for the photomorphogenesis-related alternative splicing shifts primarily controlled by a metabolic photosynthesis-derived signal and exogenous sucrose supply, correlated with the expression of dark-induced genes under the control of SnRK1 (Hartmann et al., 2016). *AtTZF1/AtCTH/AtC3H23* (a tandem-arrayed CCCH-type zinc finger motif involved in stress‐ and hormone-mediated growth), was also identified as a sugar-sensitive gene in *Arabidopsis* (Qu et al., 2014). AtTZF1 can traffick between the nucleus and cytoplasmic foci and bind both DNA and RNA *in vitro*; it may be involved in RNA regulation and under the control of sugar signaling (Pomeranz et al., 2010). However, the basic molecular mechanisms behind this regulation have not been addressed to date.

## Conclusion

Post-transcriptional regulation is an essential component of gene expression regulation in plants. Numerous findings have unveiled and characterized various factors involved in post-transcriptional regulation. The present review provides a first comprehensive picture of the relationship between sugar (metabolism and signaling) and post-transcriptional regulation factors in plants, including RBPs, miRNAs, and mRNA stability of sugar-related genes. More work needs to be carried out to figure out the functions and mechanisms related to the involvement of post-transcriptional regulation in sugar-related processes, e.g., whether regulatory mechanisms found in human cells or yeast are also conserved in plants. Considering the frequently observed connection between mRNA abundance and sugar, some recently developed technologies for RNA editing (CRISPR-Cas13), RNA binding (RNA interactome capture, photoactivatable ribonucleoside-enhanced crosslinking), and RNA folding (DMS-seq, SHAPE-seq) will support future studies. Besides, many aspects of RNA decay still need to be studied in depth, such as the spliceosome and the editosome (a large multi-protein complex that catalyzes RNA editing), which play a crucial role in post-transcriptional regulation. Although some reports about the interaction between sugar-related RBPs and miRNAs exist, further investigations are still required to gain a comprehensive understanding of the way sugar signaling operates through each of these post-transcriptional regulation mechanisms and how they crosstalk to regulate plant growth and development. The hub role of hexokinase, SnRK1, and/or TOR kinase but also the relevance of the trehalose signaling pathway in the different post-transcriptional regulation networks could be two main future lines of research. This further knowledge will also pave the way for discovering a new and complex sugar regulatory network in plants.

## Author Contributions

All authors listed have made direct contribution to the work, and approved it for publication. MW, LZ, FJ, SS, and JC have written different part of the manuscript. M-DP-G, LO, and LH have contributed to the section sugar and RNA binding proteins and JL to the section of sugars and miRNA.

### Conflict of Interest

The authors declare that the research was conducted in the absence of any commercial or financial relationships that could be construed as a potential conflict of interest.

## References

[ref1] Arroyo A. Bossi F. Finkelstein R. R. León P. (2003). Three genes that affect sugar sensing (abscisic acid insensitive 4, abscisic acid insensitive 5, and constitutive triple response 1) are differentially regulated by glucose in *Arabidopsis*. Plant Physiol. 133, 231–242. 10.1104/pp.103.021089, PMID: 12970489 PMC196600

[ref3] Baena-González E. Rolland R. Thevelein J. M. Sheen J. (2007). A central integrator of transcription networks in plantstress and energy signaling. Nature 448, 938–942. 10.1038/nature06069, PMID: 17671505

[ref5] Bartel D. P. (2004). MicroRNAs: genomics, biogenesis, mechanism, and function. Cell 116, 281–297. 10.1016/S0092-8674(04)00045-5, PMID: 14744438

[ref6] Bartel D. P. (2018). Metazoan micrornasmicroRNAs. Cell 173, 20–51. 10.1016/j.cell.2018.03.006 29570994 PMC6091663

[ref7] Berardini T. Z. Reiser L. Li D. Mezheritsky Y. Muller R. Strait E.. (2015). The *Arabidopsis* information resource: making and mining the “gold standard” annotated reference plant genome. Genesis 53, 474–485. 10.1002/dvg.22877, PMID: 26201819 PMC4545719

[ref8] Bhogale S. Mahajan A. S. Natarajan B. Rajabhoj M. Thulasiram H. V. Banerjee A. K. (2014). MicroRNA156: a potential graft-transmissible microRNA that modulates plant architecture and tuberization in *Solanum tuberosum ssp. andigena*. Plant Physiol. 164, 1011–1027. 10.1104/pp.113.230714, PMID: 24351688 PMC3912076

[ref9] Bi C. Ma Y. Jiang S. C. Mei C. Wang X. F. Zhang D. P. (2019). *Arabidopsis* translation initiation factors eIF iso4G1/2 link repression of mRNA cap-binding complex eIF iso4F assembly with RNA-binding protein SOAR 1-mediated ABA signaling. New Phytol. 223, 1388–1406. 10.1111/nph.15880, PMID: 31050354

[ref10] Braun D. M. Wang L. Ruan Y. L. (2014). Understanding and manipulating sucrose phloem loading, unloading, metabolism, and signalling to enhance crop yield and food security. J. Exp. Bot. 65, 1713–1735. 10.1093/jxb/ert416, PMID: 24347463

[ref11] Cai Q. Liang C. Wang S. Hou Y. Gao L. Liu L.. (2018). The disease resistance protein SNC1 represses the biogenesis of microRNAs and phased siRNAs. Nat. Commun. 9:5080. 10.1038/s41467-018-07516-z, PMID: 30498229 PMC6265325

[ref12] Carrió-Seguí À. Ruiz-Rivero O. Villamayor-Belinchón L. Puig S. Perea-García A. Peñarrubia L. (2019). The altered expression of microRNA408 influences the *Arabidopsis* response to iron deficiency. Front. Plant Sci. 10:324. 10.3389/fpls.2019.00324, PMID: 31001291 PMC6454987

[ref13] Carvalho R. F. Carvalho S. D. Duque P. (2010). The plant-specific SR45 protein negatively regulates glucose and ABA signaling during early seedling development in *Arabidopsis*. Plant Physiol. 154, 772–783. 10.1104/pp.110.155523, PMID: 20699397 PMC2949030

[ref14] Carvalho R. F. Szakonyi D. Simpson C. G. Barbosa I. C. Brown J. W. Baena-González E.. (2016). The *Arabidopsis* SR45 splicing factor, a negative regulator of sugar signaling, modulates SNF1-related protein kinase 1 stability. Plant Cell 28, 1910–1925. 10.1105/tpc.16.00301, PMID: 27436712 PMC5006706

[ref15] Chan M. T. Yu S. M. (1998). The 3' untranslated region of a rice α-amylase gene functions as a sugar-dependent mRNA stability determinant. Proc. Natl. Acad. Sci. U. S. A. 95, 6543–6547. 10.2307/45417, PMID: 9601003 PMC27866

[ref16] Chen C. Y. A. Shyu A. B. (1995). AU-rich elements: characterization and importance in mRNA degradation. Trends Biochem. Sci. 20, 465–470. 10.1016/S0968-0004(00)89102-1, PMID: 8578590

[ref17] Cheng W. H. Taliercio E. W. Chourey P. S. (1999). Sugars modulate an unusual mode of control of the cell-wall invertase gene (Incw1) through its 3' untranslated region in a cell suspension culture of maize. Proc. Natl. Acad. Sci. U. S. A. 96, 10512–10517. 10.1073/pnas.96.18.10512, PMID: 10468640 PMC17920

[ref18] Chou H. L. Tian L. Kumamaru T. Hamada S. Okita T. W. (2017). Multifunctional RNA binding protein OsTudor-SN in storage protein mRNA transport and localization. Plant Physiol. 175, 1608–1623. 10.1104/pp.17.01388, PMID: 29084903 PMC5717745

[ref19] Confraria A. Martinho C. S. D. S. Elias A. Rubio-Somoza I. Baena-González E. (2013). miRNAs mediate SnRK1-dependent energy signaling in *Arabidopsis*. Front. Plant Sci. 4:197. 10.3389/fpls.2013.00197, PMID: 23802004 PMC3687772

[ref20] Corradetti M. N. Guan K. (2006). Upstream of the mammalian target of rapamycin: do all roads pass through mTOR? Oncogene 25, 6347–6360. 10.1038/sj.onc.1209885, PMID: 17041621

[ref21] Dekkers B. J. Schuurmans J. A. Smeekens S. C. (2008). Interaction between sugar and abscisic acid signalling during early seedling development in *Arabidopsis*. Plant Mol. Biol. 67, 151–167. 10.1007/s11103-008-9308-6, PMID: 18278579 PMC2295253

[ref22] Delatte T. L. Sedijani P. Kondou Y. Matsui M. de Jong G. J. Somsen G. W.. (2011). Growth arrest by trehalose-6-phosphate: an astonishing case of primary metabolite control over growth by way of the SnRK1 signaling pathway. Plant Physiol. 157, 160–174. 10.1104/pp.111.180422, PMID: 21753116 PMC3165867

[ref23] Dobrenel T. Caldana C. Hanson J. Robaglia C. Vincentz M. Veit B.. (2016). TOR signaling and nutrient sensing. Annu. Rev. Plant Biol. 67, 261–285. 10.1146/annurev-arplant-043014-114648, PMID: 26905651

[ref24] Doidy J. Vidal U. Lemoine R. (2019). Sugar transporters in Fabaceae, featuring SUT MST and SWEET families of the model plant *Medicago truncatula* and the agricultural crop *Pisum sativum*. PLoS One 14:e0223173. 10.1371/journal.pone.0223173, PMID: 31568488 PMC6768477

[ref25] Duarte G. T. Matiolli C. C. Pant B. D. Schlereth A. Scheible W. R. Stitt M.. (2013). Involvement of microRNA-related regulatory pathways in the glucose-mediated control of *Arabidopsis* early seedling development. J. Exp. Bot. 64, 4301–4312. 10.1093/jxb/ert239, PMID: 23997203 PMC3808316

[ref26] Dugas D. V. Bartel B. (2008). Sucrose induction of *Arabidopsis* miR398 represses two Cu/Zn superoxide dismutases. Plant Mol. Biol. 67, 403–417. 10.1007/s11103-008-9329-1, PMID: 18392778

[ref27] Farooq M. A. Niazi A. K. Akhtar J. Saifullah U. Farooq M. Souri Z.. (2019). Acquiring control: the evolution of ROS-induced oxidative stress and redox signaling pathways in plant stress responses. Plant Physiol. Biochem. 141, 353–369. 10.1016/j.plaphy.2019.04.039, PMID: 31207496

[ref28] Feldbrügge M. Arizti P. Sullivan M. L. Zamore P. D. Belasco J. G. Green P. J. (2002). Comparative analysis of the plant mRNA-destabilizing element, DST, in mammalian and tobacco cells. Plant Mol. Biol. 49, 215–223. 10.1023/a:1014936824187, PMID: 11999376

[ref29] Feng C. Chen Y. Wang C. Kong Y. Wu W. Chen Y. (2014). *Arabidopsis* RAV1 transcription factor, phosphorylated by SnRK2 kinases, regulates the expression of *ABI3*, *ABI4*, and *ABI5* during seed germination and early seedling development. Plant J. 80, 654–668. 10.1111/tpj.12670, PMID: 25231920

[ref30] Feng J. X. Liu D. Pan Y. Gong W. Ma L. G. Luo J. C.. (2005). An annotation update via cDNA sequence analysis and comprehensive profiling of developmental, hormonal or environmental responsiveness of the *Arabidopsis* AP2/EREBP transcription factor gene family. Plant Mol. Biol. 59, 853–868. 10.1007/s11103-005-1511-0, PMID: 16307362

[ref31] Feng J. Wang J. Fan P. Jia W. Nie L. Jiang P.. (2015). High-throughput deep sequencing reveals that microRNAs play important roles in salt tolerance of euhalophyte *Salicornia europaea*. BMC Plant Biol. 15:63. 10.1186/s12870-015-0451-3, PMID: 25848810 PMC4349674

[ref32] Figueroa C. M. Lunn J. E. (2016). A tale of two sugars: trehalose 6-phosphate and sucrose. Plant Physiol. 172, 7–27. 10.1104/pp.16.00417, PMID: 27482078 PMC5074632

[ref33] Flores-Pérez Ú. Pérez-Gil J. Closa M. Wright L. P. Botella-Pavía P. Phillips M. A.. (2010). Pleiotropic regulatory locus 1 (PRL1) integrates the regulation of sugar responses with isoprenoid metabolism in *Arabidopsis*. Mol. Plant 3, 101–112. 10.1093/mp/ssp100, PMID: 20008452

[ref34] Francischini C. W. Quaggio R. B. (2009). Molecular characterization of *Arabidopsis thaliana* PUF proteins–binding specificity and target candidates. FEBS J. 276, 5456–5470. 10.1111/j.1742-4658.2009.07230.x, PMID: 19682068

[ref35] Francisco-Mangilet A. G. Karlsson P. Kim M. H. Eo H. J. Oh S. A. Kim J. H.. (2015). THO_2_, a core member of the THO/TREX complex, is required for micro RNA production in *Arabidopsis*. Plant J. 82, 1018–1029. 10.1111/tpj.12874, PMID: 25976549

[ref36] Fujiki Y. Ito M. Nishida I. Watanabe A. (2000). Multiple signaling pathways in gene expression during sugar starvation. Pharmacological analysis of din gene expression in suspension-cultured cells of *Arabidopsis*. Plant Physiol. 124, 1139–1148. 10.1104/pp.124.3.1139, PMID: 11080291 PMC59213

[ref37] Fusaro A. F. Bocca S. N. Ramos R. L. B. Barrôco R. M. Magioli C. Jorge V. C.. (2007). AtGRP2, a cold-induced nucleo-cytoplasmic RNA-binding protein, has a role in flower and seed development. Planta 225, 1339–1351. 10.1007/s00425-006-0444-4, PMID: 17123099

[ref38] Gao R. Wang Y. Gruber M. Y. Hannoufa A. (2018). miR156/SPL10 modulates lateral root development, branching and leaf morphology in *Arabidopsis* by silencing AGAMOUS-LIKE 79. Front. Plant Sci. 8:2226. 10.3389/fpls.2017.02226, PMID: 29354153 PMC5758603

[ref39] Garapati P. Feil R. Lunn J. E. Van Dijck P. Balazadeh S. Mueller-Roeber M. (2015). Transcription factor *Arabidopsis* activating factor1 integrates carbon starvation responses with trehalose metabolism. Plant Physiol. 169, 379–390. 10.1104/pp.15.00917, PMID: 26149570 PMC4577426

[ref40] Gilmonreal M. Zabalza A. Missihoun T. D. Dormann P. Bartels D. Royuela M. (2017). Induction of the PDH bypass and upregulation of the ALDH7B4 in plants treated with herbicides inhibiting amino acid biosynthesis. Plant Sci. 264, 16–28. 10.1016/j.plantsci.2017.08.003, PMID: 28969796

[ref41] Granot D. Kelly G. Stein O. David-Schwartz R. (2014). Substantial roles of hexokinase and fructokinase in the effects of sugars on plant physiology and development. J. Exp. Bot. 65, 809–819. 10.1093/jxb/ert400, PMID: 24293612

[ref42] Guerra D. Crosatti C. Khoshro H. H. Mastrangelo A. M. Mica E. Mazzucotelli E. (2015). Post-transcriptional and post-translational regulations of drought and heat response in plants: a spider’s web of mechanisms. Front. Plant Sci. 6:57. 10.3389/fpls.2015.00057, PMID: 25717333 PMC4324062

[ref43] Hartmann L. Drewe-Boß P. Wießner T. Wagner G. Geue S. Lee H. C.. (2016). Alternative splicing substantially diversifies the transcriptome during early photomorphogenesis and correlates with the energy availability in *Arabidopsis*. Plant Cell 28, 2715–2734. 10.1105/tpc.16.00508, PMID: 27803310 PMC5155347

[ref44] He H. Chincinska I. Hackel A. Grimm B. Kühn C. (2008). Phloem mobility and stability of sucrose transporter transcripts. Open Plant Sci. J. 2, 1–14. 10.2174/1874294700802010001

[ref45] Ho S. L. Chao Y. C. Tong W. F. Yu S. M. (2001). Sugar coordinately and differentially regulates growth-and stress-related gene expression via a complex signal transduction network and multiple control mechanisms. Plant Physiol. 125, 877–890. 10.1104/pp.125.2.877, PMID: 11161045 PMC64889

[ref46] Hou Q. Bartels D. (2015). Comparative study of the aldehyde dehydrogenase (ALDH) gene superfamily in the glycophyte *Arabidopsis thaliana* and *Eutrema halophytes*. Ann. Bot. 115, 465–479. 10.1093/aob/mcu152, PMID: 25085467 PMC4332599

[ref47] Hu J. Liu Y. Tang X. Rao H. Pei J. (2020). Transcriptome profiling of the flowering transition in saffron (*Crocus sativus* L.). Sci. Rep. 10:9680. 10.1038/s41598-020-66675-6, PMID: 32541892 PMC7295807

[ref48] Hu J. Y. Zhou Y. He F. Dong X. Liu L. Y. Coupland G.. (2014). miR824-regulated AGAMOUS-LIKE16 contributes to flowering time repression in *Arabidopsis*. Plant Cell 26, 2024–2037. 10.1105/tpc.114.124685, PMID: 24876250 PMC4079366

[ref49] Huang L. F. Bocock P. N. Davis J. M. Koch K. E. (2007). Regulation of invertase: a ‘suite’ of transcriptional and post-transcriptional mechanisms. Funct. Plant Biol. 34, 499–507. 10.1071/fp06227, PMID: 32689379

[ref50] Huo X. Wang C. Teng Y. Liu X. (2015). Identification of miRNAs associated with dark-induced senescence in *Arabidopsis*. BMC Plant Biol. 15:266. 10.1186/s12870-015-0656-5, PMID: 26530097 PMC4632659

[ref51] Hwang G. Kim S. Cho J. Y. Paik I. Kim J. I. Oh E. (2019). Trehalose-6-phosphate signaling regulates thermoresponsive hypocotyl growth in *Arabidopsis thaliana*. EMBO Rep. 20:e47828. 10.15252/embr.201947828, PMID: 31393060 PMC6776909

[ref52] Jang Y. H. Park H. Y. Kim S. K. Lee J. H. Suh M. C. Chung Y. S.. (2009). Survey of rice proteins interacting with OsFCA and OsFY proteins which are homologous to the *Arabidopsis* flowering time proteins, FCA and FY. Plant Cell Physiol. 50, 1479–1492. 10.1093/pcp/pcp093, PMID: 19561057

[ref53] Janse van Rensburg H. C. Van den Ende W. (2018). UDP-glucose: a potential signaling molecule in plants? Front. Plant Sci. 8:2230. 10.3389/fpls.2017.02230, PMID: 29375604 PMC5767297

[ref54] Jia X. Wang W. Ren L. Chen Q. Mendu V. Willcut B.. (2009). Differential and dynamic regulation of miR398 in response to ABA and salt stress in *Populus tremula* and *Arabidopsis thaliana*. Plant Mol. Biol. 71, 51–59. 10.1007/s11103-009-9508-8, PMID: 19533381

[ref55] Jia T. Zhang B. You C. Zhang Y. Zeng L. Li S.. (2017). The *Arabidopsis* MOS4-associated complex promotes microRNA biogenesis and precursor messenger RNA splicing. Plant Cell 29, 2626–2643. 10.1105/tpc.17.00370, PMID: 28947490 PMC5774577

[ref56] Jiang S. C. Mei C. Liang S. Yu Y. T. Lu K. Wu Z.. (2015). Crucial roles of the pentatricopeptide repeat protein SOAR1 in *Arabidopsis* response to drought, salt and cold stresses. Plant Mol. Biol. 88, 369–385. 10.1007/s11103-015-0327-9, PMID: 26093896 PMC4486114

[ref58] Jiao Y. Lei W. Xu W. Chen W. L. (2019). Glucose signaling, AtRGS1 and plant autophagy. Plant Signal. Behav. 14:1607465. 10.1080/15592324.2019.1607465, PMID: 31055999 PMC6619941

[ref59] Jiao Y. Wang Y. Xue D. Wang J. Yan M. Liu G.. (2020). Regulation of OsSPL14 by OsmiR156 defines ideal plant architecture in rice. Nat. Genet. 42, 541–544. 10.1038/ng.591, PMID: 20495565

[ref60] Jung J. H. Seo P. J. Ahn J. H. Park C. M. (2012). *Arabidopsis* RNA-binding protein FCA regulates microRNA172 processing in thermosensory flowering. J. Biol. Chem. 287, 16007–16016. 10.1074/jbc.M111.337485, PMID: 22431732 PMC3346135

[ref61] Kanwar P. Jha G. (2019). Alterations in plant sugar metabolism: signatory of pathogen attack. Planta 249, 305–318. 10.1007/s00425-018-3018-3, PMID: 30267150

[ref62] Karlsson P. Christie M. D. Seymour D. K. Wang H. Wang X. Hagmann J.. (2015). KH domain protein RCF3 is a tissue-biased regulator of the plant miRNA biogenesis cofactor HYL1. Proc. Natl. Acad. Sci. U. S. A. 112, 14096–14101. 10.1073/pnas.1512865112, PMID: 26512101 PMC4653147

[ref63] Kawa D. Testerink C. (2017). Regulation of mRNA decay in plant responses to salt and osmotic stress. Cell. Mol. Life Sci. 74, 1165–1176. 10.1007/s00018-016-2376-x, PMID: 27677492 PMC5346435

[ref64] Kesarwani A. K. Lee H. C. Ricca P. G. Sullivan G. Faiss N. Wagner G.. (2019). Multifactorial and species-specific feedback regulation of the RNA surveillance pathway nonsense-mediated decay in plants. Plant Cell Physiol. 60, 1986–1999. 10.1093/pcp/pcz141, PMID: 31368494

[ref65] Kim Y. O. Kang H. (2006). The role of a zinc finger-containing glycine-rich RNA-binding protein during the cold adaptation process in *Arabidopsis thaliana*. Plant Cell Physiol. 47, 793–798. 10.1093/pcp/pcj047, PMID: 16608866

[ref66] Kim J. J. Lee J. H. Kim W. Jung H. S. Huijser P. Ahn J. H. (2012). The microRNA156-SQUAMOSA PROMOTER BINDING PROTEIN-LIKE3 module regulates ambient temperature-responsive flowering via FLOWERING LOCUS T in *Arabidopsis*. Plant Physiol. 159, 461–478. 10.1104/pp.111.192369, PMID: 22427344 PMC3375978

[ref67] Kim Y. O. Pan S. Jung C. H. Kang H. (2007b). A zinc finger-containing glycine-rich RNA-binding protein, atRZ-1a, has a negative impact on seed germination and seedling growth of *Arabidopsis thaliana* under salt or drought stress conditions. Plant Cell Physiol. 48, 1170–1181. 10.1093/pcp/pcm087, PMID: 17602187

[ref68] Kim J. Y. Park S. J. Jang B. Jung C. H. Ahn S. J. Goh C. H.. (2007a). Functional characterization of a glycine-rich RNA-binding protein 2 in *Arabidopsis thaliana* under abiotic stress conditions. Plant J. 50, 439–451. 10.1111/j.1365-313X.2007.03057.x, PMID: 17376161

[ref69] Kirch H. H. Schlingensiepen S. Kotchoni S. Sunkar R. Bartels D. (2005). Detailed expression analysis of selected genes of the aldehyde dehydrogenase (ALDH) gene superfamily in *Arabidopsis thaliana*. Plant Mol. Biol. 57, 315–332. 10.1007/s11103-004-7796-6, PMID: 15830124

[ref70] Kohorn B. D. Johansen S. Shishido A. Todorova T. Martinez R. Defeo E.. (2009). Pectin activation of MAP kinase and gene expression is WAK2 dependent. Plant J. 60, 974–982. 10.1111/j.1365-313X.2009.04016.x, PMID: 19737363 PMC3575133

[ref71] Kohorn B. D. Kobayashi M. Johansen S. Riese J. Huang L. F. Koch K.. (2006). An *Arabidopsis* cell wall-associated kinase required for invertase activity and cell growth. Plant J. 46, 307–316. 10.1111/j.1365-313X.2006.02695.x, PMID: 16623892

[ref72] Koyama T. Sato F. Ohme-Takagi M. (2017). Roles of miR319 and TCP transcription factors in leaf development. Plant Physiol. 175, 874–885. 10.1104/pp.17.00732, PMID: 28842549 PMC5619901

[ref162] Kühn C. Grof C. P. L. (2010). Sucrose transporters of higher plants. Curr. Opin. Plant Biol. 13, 287–297. 10.1016/j.pbi.2010.02.001, PMID: 20303321

[ref73] Kühn C. Franceschi V. R. Schulz A. Lemoine R. Frommer W. B. (1997). Macromolecular trafficking indicated by localization and turnover of sucrose transporters in enucleate sieve elements. Science 275, 1298–1300. 10.1126/science.275.5304.1298, PMID: 9036853

[ref74] Kumar A. Kondhare K. R. Vetal P. V. Banerjee A. K. (2020). PcG proteins MSI1 and BMI1 function upstream of miR156 to regulate aerial tuber formation in potato. Plant Physiol. 182, 185–203. 10.1104/pp.19.00416, PMID: 31427464 PMC6945842

[ref75] Lastdrager J. Hanson J. Smeekens S. (2014). Sugar signals and the control of plant growth and development. J. Exp. Bot. 65, 799–807. 10.1093/jxb/ert474, PMID: 24453229

[ref76] Lee K. Kang H. (2016). Emerging roles of RNA-binding proteins in plant growth, development, and stress responses. Mol. Cell 39, 179–185. 10.14348/molcells.2016.2359, PMID: 26831454 PMC4794599

[ref77] Li X. Cai W. Liu Y. Li H. Fu L. Liu Z.. (2017). Differential TOR activation and cell proliferation in *Arabidopsis* root and shoot apexes. Proc. Natl. Acad. Sci. U. S. A. 114, 2765–2770. 10.1073/pnas.1618782114, PMID: 28223530 PMC5347562

[ref78] Li L. Sheen J. (2016). Dynamic and diverse sugar signaling. Curr. Opin. Plant Biol. 33, 116–125. 10.1016/j.pbi.2016.06.018, PMID: 27423125 PMC5050104

[ref79] Liesche J. Krügel U. He H. Chincinska I. Hackel A. Kühn C. (2011). Sucrose transporter regulation at the transcriptional, post-transcriptional and post-translational level. J. Plant Physiol. 168, 1426–1433. 10.1016/j.jplph.2011.02.005, PMID: 21444123

[ref80] Lin J. S. Kuo C. C. Yang I. C. Tsai W. A. Shen Y. H. Lin C. C.. (2018). MicroRNA160 modulates plant development and heat shock protein gene expression to mediate heat tolerance in *Arabidopsis*. Front. Plant Sci. 9:68. 10.3389/fpls.2018.00068, PMID: 29449855 PMC5799662

[ref81] Liu Y. Bassham D. C. (2010). TOR is a negative regulator of autophagy in *Arabidopsis thaliana*. PLoS One 5:e11883. 10.1371/journal.pone.0011883, PMID: 20686696 PMC2912371

[ref83] Liu F. Quesada V. Crevillén P. Bäurle I. Swiezewski S. Dean C. (2007). The *Arabidopsis* RNA-binding protein FCA requires a lysine-specific demethylase 1 homolog to downregulate FLC. Mol. Cell 28, 398–407. 10.1016/j.molcel.2007.10.018, PMID: 17996704

[ref84] Lorković Z. J. (2009). Role of plant RNA-binding proteins in development, stress response and genome organization. Trends Plant Sci. 14, 229–236. 10.1016/j.tplants.2009.01.007, PMID: 19285908

[ref85] Lu Y. Sun J. Yang Z. Zhao C. Zhu M. Ma D.. (2019). Genome-wide identification and expression analysis of glycine-rich RNA-binding protein family in sweet potato wild relative *Ipomoea trifida*. Gene 686, 177–186. 10.1016/j.gene.2018.11.044, PMID: 30453066

[ref86] Macknight R. Bancroft I. Page T. Lister C. Schmidt R. Love K.. (1997). FCA, a gene controlling flowering time in *Arabidopsis*, encodes a protein containing RNA-binding domains. Cell 89, 737–745. 10.1016/S0092-8674(00)80256-1, PMID: 9182761

[ref87] Mahalingam R. Walling J. G. (2020). Genomic survey of RNA recognition motif (RRM) containing RNA binding proteins from barley (*Hordeum vulgare ssp. vulgare*). Genomics 112, 1829–1839. 10.1016/j.ygeno.2019.10.016, PMID: 31669702

[ref88] Manavella P. A. Hagmann J. Ott F. Laubinger S. Franz M. Macek B.. (2012). Fast-forward genetics identifies plant CPL phosphatases as regulators of miRNA processing factor HYL1. Cell 151, 859–870. 10.1016/j.cell.2012.09.039, PMID: 23141542

[ref163] Marondedze C. Thomas L. Serrano N. L. Lilley K. S. Gehring C. (2016). The RNA-binding protein repertoire of *Arabidopsis thaliana*. Sci. Rep. 6:29766. 10.1038/srep29766, PMID: 27405932 PMC4942612

[ref89] Marondedze C. Thomas L. Gehring C. Lilley K. S. (2019). Changes in the *Arabidopsis* RNA-binding proteome reveal novel stress response mechanisms. BMC Plant Biol. 19:139. 10.1186/s12870-019-1750-x, PMID: 30975080 PMC6460520

[ref90] Martin R. C. Asahina M. Liu P. Kristof J. R. Coppersmith J. L. Pluskota W. E. (2010). The microRNA156 and microRNA172 gene regulation cascades at post-germinative stages in *Arabidopsis*. Seed Sci. Res. 20, 79–87. 10.1017/s0960258510000085

[ref91] Matiolli C. C. Tomaz J. P. Duarte G. T. Prado F. M. Del Bem L. E. V. Silveira A. B.. (2011). The *Arabidopsis* bZIP gene AtbZIP63 is a sensitive integrator of transient abscisic acid and glucose signals. Plant Physiol. 157, 692–705. 10.1104/pp.111.181743, PMID: 21844310 PMC3192551

[ref92] Mei C. Jiang S. C. Lu Y. F. Wu F. Q. Yu Y. T. Liang S.. (2014). *Arabidopsis* pentatricopeptide repeat protein SOAR1 plays a critical role in abscisic acid signalling. J. Exp. Bot. 65, 5317–5330. 10.1093/jxb/eru293, PMID: 25005137 PMC4157714

[ref93] Meng L. Xu M. Wan W. Yu F. Li C. Wang J.. (2018). Sucrose signaling regulates anthocyanin biosynthesis through a MAPK cascade in *Arabidopsis thaliana*. Genetics 210, 607–619. 10.1534/genetics.118.301470, PMID: 30143593 PMC6216600

[ref94] Meyers B. C. Axtell M. J. (2019). MicroRNAs in plants: key findings from the early years. Plant Cell 31:1206. 10.1105/tpc.19.00310, PMID: 31036598 PMC6588298

[ref95] Michaels S. D. Amasino R. M. (1999). FLOWERING LOCUS C encodes a novel MADS domain protein that acts as a repressor of flowering. Plant Cell 11, 949–956. 10.1105/tpc.11.5.949, PMID: 10330478 PMC144226

[ref96] Milne R. J. Perroux J. M. Rae A. L. Reinders A. Ward J. M. Offler C. E.. (2017). Sucrose transporter localization and function in phloem unloading in developing stems. Plant Physiol. 173, 1330–1341. 10.1104/pp.16.01594, PMID: 27986867 PMC5291036

[ref97] Nagarajan V. K. Kukulich P. M. von Hagel B. Green P. J. (2019). RNA degradomes reveal substrates and importance for dark and nitrogen stress responses of Arabidopsis XRN4. Nucleic Acids Res. 47, 9216–9230. 10.1093/nar/gkz712, PMID: 31428786 PMC6755094

[ref98] Nicolai M. Roncato M. A. Canoy A. S. Rouquie D. Sarda X. Freyssinet G.. (2006). Large-scale analysis of mRNA translation states during sucrose starvation in *Arabidopsis* cells identifies cell proliferation and chromatin structure as targets of translational control. Plant Physiol. 141, 663–673. 10.1104/pp.106.079418, PMID: 16632591 PMC1475480

[ref99] Pan J. Huang D. Guo Z. Kuang Z. Zhang H. Xie X.. (2018). Overexpression of microRNA408 enhances photosynthesis, growth, and seed yield in diverse plants. J. Integr. Plant Biol. 60, 323–340. 10.1111/jipb.12634, PMID: 29330900

[ref100] Pant B. D. Musialak-Lange M. Nuc P. May P. Buhtz A. Kehr J.. (2009). Identification of nutrient-responsive *Arabidopsis* and rapeseed microRNAs by comprehensive real-time polymerase chain reaction profiling and small RNA sequencing. Plant Physiol. 150, 1541–1555. 10.1104/pp.109.139139, PMID: 19465578 PMC2705054

[ref101] Park S. Y. Grabau E. (2017). Bypassing miRNA-mediated gene regulation under drought stress: alternative splicing affects CSD1 gene expression. Plant Mol. Biol. 95, 243–252. 10.1007/s11103-017-0642-4, PMID: 28776286

[ref102] Pomeranz M. C. Hah C. Lin P. C. Kang S. G. Finer J. J. Blackshear P. J.. (2010). The *Arabidopsis* tandem zinc finger protein AtTZF1 traffics between the nucleus and cytoplasmic foci and binds both DNA and RNA. Plant Physiol. 152, 151–165. 10.1104/pp.109.145656, PMID: 19897605 PMC2799353

[ref103] Ponnu J. Schlereth A. Zacharaki V. Działo M. A. Abel C. Feil R.. (2020). The trehalose 6-phosphate pathway impacts vegetative phase change in *Arabidopsis thaliana*. Plant J. 10.1111/tpj.14965, PMID: [Epub ahead of print] 32799402

[ref104] Qu J. Kang S. G. Wang W. Musier-Forsyth K. Jang J. C. (2014). The *Arabidopsis thaliana* tandem zinc finger 1 (AtTZF1) protein in RNA binding and decay. Plant J. 78, 452–467. 10.1111/tpj.12485, PMID: 24635033 PMC4026020

[ref105] Reichel M. Liao Y. Rettel M. Ragan C. Evers M. Alleaume A. M.. (2016). In planta determination of the mRNA-binding proteome of *Arabidopsis* etiolated seedlings. Plant Cell 28, 2435–2452. 10.1105/tpc.16.00562, PMID: 27729395 PMC5134986

[ref106] Ren L. Tang G. (2012). Identification of sucrose-responsive microRNAs reveals sucrose-regulated copper accumulations in an SPL7-dependent and independent manner in *Arabidopsis thaliana*. Plant Sci. 187, 59–68. 10.1016/j.plantsci.2012.01.014, PMID: 22404833

[ref107] Ren G. Xie M. Dou Y. Zhang S. Zhang C. Yu B. (2012). Regulation of miRNA abundance by RNA binding protein TOUGH in *Arabidopsis*. Proc. Natl. Acad. Sci. U. S. A. 109, 12817–12821. 10.1073/pnas.1204915109, PMID: 22802657 PMC3412041

[ref108] Riechmann J. L. Heard J. Martin G. Reuber L. Jiang C. -Z. Keddie J.. (2000). *Arabidopsis* transcription factors: genome-wide comparative analysis among eukaryotes. Science 290, 2105–2110. 10.1126/science.290.5499.2105, PMID: 11118137

[ref109] Rigo R. Bazin J. Crespi M. Charon C. (2019). Alternative splicing in the regulation of plant–microbe interactions. Plant Cell Physiol. 60, 1906–1916. 10.1093/pcp/pcz086, PMID: 31106828

[ref110] Rodriguez M. Parola R. Andreola S. Pereyra C. Martínez-Noël G. (2019). TOR and SnRK1 signaling pathways in plant response to abiotic stresses: do they always act according to the “yin-yang” model? Plant Sci. 288:110220. 10.1016/j.plantsci.2019.110220, PMID: 31521220

[ref111] Rogers K. Chen X. (2013). Biogenesis, turnover, and mode of action of plant microRNAs. Plant Cell 25, 2383–2399. 10.1105/tpc.113.113159, PMID: 23881412 PMC3753372

[ref112] Romanowski A. Yanovsky M. J. (2015). Circadian rhythms and post-transcriptional regulation in higher plants. Front. Plant Sci. 6:437. 10.3389/fpls.2015.00437, PMID: 26124767 PMC4464108

[ref113] Rosenberger C. L. Chen J. (2018). To grow or not to grow: TOR and SnRK2 coordinate growth and stress response in *Arabidopsis*. Mol. Cell 69, 3–4. 10.1016/j.molcel.2017.12.013, PMID: 29304332

[ref114] Sakr S. Wang M. Dédaldéchamp F. Perez-Garcia M. D. Ogé L. Hamama L.. (2018). The sugar-signaling hub: overview of regulators and interaction with the hormonal and metabolic network. Int. J. Mol. Sci. 19:2506. 10.3390/ijms19092506, PMID: 30149541 PMC6165531

[ref164] Samad A. F. A. Sajad M. Nazaruddin N. Fauzi I. A. Murad A. Murad A. M. A.. (2017). MicroRNA and transcription factor: key players in plant regulatory network. Front. Plant Sci. 8:565. 10.3389/fpls.2017.00565, PMID: 28446918 PMC5388764

[ref115] Sami F. Siddiqui H. Hayat S. (2019). Interaction of glucose and phytohormone signaling in plants. Plant Physiol. Biochem. 135, 119–126. 10.1016/j.plaphy.2018.11.005, PMID: 30529977

[ref116] Sheu J. J. Jan S. P. Lee H. T. Yu S. M. (1994). Control of transcription and mRNA turnover as mechanisms of metabolic repression of α-amylase gene expression. Plant J. 5, 655–664. 10.1111/j.1365-313x.1994.00655.x

[ref117] Shi L. Wu Y. Sheen J. (2018). TOR signaling in plants: conservation and innovation. Development 145:dev160887. 10.1242/dev.160887, PMID: 29986898 PMC6053665

[ref118] Shinozawa A. Otake R. Takezawa D. Umezawa T. Komatsu K. Tanaka K.. (2019). SnRK2 protein kinases represent an ancient system in plants for adaptation to a terrestrial environment. Commun. Biol. 2:30. 10.1038/s42003-019-0281-1, PMID: 30675528 PMC6340887

[ref119] Signorelli S. Masclaux-Daubresse C. Moriyasu Y. Van den Ende W. Bassham D. C. (2019). Sugars and autophagy in plants. Front. Plant Sci. 10:1190. 10.3389/fpls.2019.01190, PMID: 31632424 PMC6779131

[ref120] Silverman I. M. Li F. Gregory B. D. (2013). Genomic era analyses of RNA secondary structure and RNA-binding proteins reveal their significance to post-transcriptional regulation in plants. Plant Sci. 205–206, 55–62. 10.1016/j.plantsci.2013.01.009, PMID: 23498863 PMC4079699

[ref121] Song X. Li Y. Cao X. Qi Y. (2019). MicroRNAs and their regulatory roles in plant–environment interactions. Annu. Rev. Plant Biol. 70, 489–525. 10.1146/annurev-arplant-050718-100334, PMID: 30848930

[ref122] Stitt M. Zeeman S. C. (2012). Starch turnover: pathways, regulation and role in growth. Curr. Opin. Plant Biol. 15, 282–292. 10.1016/j.pbi.2012.03.016, PMID: 22541711

[ref123] Su C. Li Z. Cheng J. Li L. Zhong S. Liu L.. (2017). The protein phosphatase 4 and SMEK1 complex dephosphorylates HYL1 to promote miRNA biogenesis by antagonizing the MAPK cascade in *Arabidopsis*. Dev. Cell 41, 527.e5–539.e5. 10.1016/j.devcel.2017.05.008, PMID: 28586645

[ref124] Sun Z. Li M. Zhou Y. Guo T. Liu Y. Zhang H.. (2018). Coordinated regulation of *Arabidopsis* microRNA biogenesis and red light signaling through Dicer-like 1 and phytochrome-interacting factor 4. PLoS Genet. 14:e1007247. 10.1371/journal.pgen.1007247, PMID: 29522510 PMC5862502

[ref125] Sunkar R. Kapoor A. Zhu J. K. (2006). Post-transcriptional induction of two Cu/Zn superoxide dismutase genes in *Arabidopsis* is mediated by downregulation of miR398 and important for oxidative stress tolerance. Plant Cell 18, 2051–2065. 10.1105/tpc.106.041673, PMID: 16861386 PMC1533975

[ref126] Swarbreck D. Wilks C. Lamesch P. Berardini T. Z. Garcia-Hernandez M. Foerster H.. (2007). The *Arabidopsis* information resource (TAIR): gene structure and function annotation. Nucleic Acids Res. 36(Suppl. 1), D1009–D1014. 10.1093/nar/gkm965, PMID: 17986450 PMC2238962

[ref127] Tian L. Chou H. L. Zhang L. Hwang S. K. Starkenburg S. R. Doroshenk K. A.. (2018a). RNA-binding protein RBP-P is required for glutelin and prolamine mRNA localization in rice endosperm cells. Plant Cell 30, 2529–2552. 10.1105/tpc.18.00321, PMID: 30190374 PMC6241268

[ref128] Tian L. Liu H. Ren L. Ku L. Wu L. Li M.. (2018b). MicroRNA 399 as a potential integrator of photo-response, phosphate homeostasis, and sucrose signaling under long day condition. BMC Plant Biol. 18:290. 10.1186/s12870-018-1460-9, PMID: 30463514 PMC6249786

[ref129] Van Leene J. Han C. Gadeyne A. Eeckhout D. Matthijs C. Cannoot B.. (2019). Capturing the phosphorylation and protein interaction landscape of the plant TOR kinase. Nat. Plants 5:316. 10.1038/s41477-019-0378-z, PMID: 30833711

[ref130] van Mourik H. van Dijk A. D. Stortenbeker N. Angenent G. C. Bemer M. (2017). Divergent regulation of *Arabidopsis* SAUR genes: a focus on the SAUR10-clade. BMC Plant Biol. 17:245. 10.1186/s12870-017-1210-4, PMID: 29258424 PMC5735953

[ref131] Voinnet O. (2009). Origin, biogenesis, and activity of plant microRNAs. Cell 136, 669–687. 10.1016/j.cell.2009.01.046, PMID: 19239888

[ref132] Wahl V. Ponnu J. Schlereth A. Arrivault S. Langenecker T. Franke A.. (2013). Regulation of flowering by trehalose-6-phosphate signaling in *Arabidopsis thaliana*. Science 339, 704–707. 10.1126/science.1230406, PMID: 23393265

[ref133] Wang J. Czech B. Weigel D. (2009). miR156-regulated SPL transcription factors define an endogenous flowering pathway in *Arabidopsis thaliana*. Cell 138, 738–749. 10.1016/j.cell.2009.06.014, PMID: 19703399

[ref134] Wang Y. Li L. Ye T. Lu Y. Chen X. Wu Y. (2013). The inhibitory effect of ABA on floral transition is mediated by ABI5 in *Arabidopsis*. J. Exp. Bot. 64, 675–684. 10.1093/jxb/ers361, PMID: 23307919 PMC3542054

[ref135] Wang M. Moigne M. L. Bertheloot J. Crespel L. Perezgarcia M. D. Oge L.. (2019b). BRANCHED1: a key hub of shoot branching. Front. Plant Sci. 10:76. 10.3389/fpls.2019.00076, PMID: 30809235 PMC6379311

[ref136] Wang M. Ogé L. Perez-Garcia M. D. Hamama L. Sakr S. (2018a). The PUF protein family: overview on PUF RNA targets, biological functions, and post-transcriptional regulation. Int. J. Mol. Sci. 19:410. 10.3390/ijms19020410, PMID: 29385744 PMC5855632

[ref137] Wang M. Ogé L. Voisine L. Perez-Garcia M. D. Jeauffre J. Hibrand Saint-Oyant L.. (2019a). Post-transcriptional regulation of *RhBRC1* (*Rosa hybrida BRANCHED1*) in response to sugars is mediated via its own 3'untranslated region, with a potential role of RhPUF4 (Pumilio RNA-binding protein family). Int. J. Mol. Sci. 20:3808. 10.3390/ijms20153808, PMID: 31382685 PMC6695800

[ref138] Wang S. Quan L. Li S. You C. Zhang Y. Gao L.. (2019c). The PROTEIN PHOSPHATASE4 complex promotes transcription and processing of primary microRNAs in *Arabidopsis*. Plant Cell 31, 486–501. 10.1105/tpc.18.00556, PMID: 30674692 PMC6447022

[ref139] Wang H. Wang H. (2015). The miR156/SPL module, a regulatory hub and versatile toolbox, gears up crops for enhanced agronomic traits. Mol. Plant 8, 677–688. 10.1016/j.molp.2015.01.008, PMID: 25617719

[ref140] Wang P. Zhao Y. Li Z. Hsu C. Liu X. Fu L.. (2018b). Reciprocal regulation of the TOR kinase and ABA receptor balances plant growth and stress response. Mol. Cell 69, 100.e6–112.e6. 10.1016/j.molcel.2017.12.002, PMID: 29290610 PMC5772982

[ref141] Wei Q. Ma C. Xu Y. Wang T. Chen Y. Lü J.. (2017). Control of chrysanthemum flowering through integration with an aging pathway. Nat. Commun. 8:829. 10.1038/s41467-017-00812-0, PMID: 29018260 PMC5635119

[ref142] Wilkinson M. E. Charenton C. Nagai K. (2020). RNA splicing by the spliceosome. Annu. Rev. Biochem. 89, 359–388. 10.1146/annurev-biochem-091719-064225, PMID: 31794245

[ref144] Wingler A. (2018). Transitioning to the next phase: the role of sugar signaling throughout the plant life cycle. Plant Physiol. 176, 1075–1084. 10.1104/pp.17.01229, PMID: 28974627 PMC5813577

[ref145] Wu G. Park M. Y. Conway S. R. Wang J. W. Weigel D. Poethig R. S. (2009). The sequential action of miR156 and miR172 regulates developmental timing in *Arabidopsis*. Cell 138, 750–759. 10.1016/j.cell.2009.06.031, PMID: 19703400 PMC2732587

[ref146] Wu X. Shi Y. Li J. Xu L. Fang Y. Li X.. (2013). A role for the RNA-binding protein MOS2 in microRNA maturation in *Arabidopsis*. Cell Res. 23, 645–657. 10.1038/cr.2013.23, PMID: 23399598 PMC3641593

[ref147] Xu M. Hu T. Zhao J. Park M. Y. Earley K. W. Wu G.. (2016). Developmental functions of miR156-regulated SQUAMOSA PROMOTER BINDING PROTEIN-LIKE (SPL) genes in *Arabidopsis thaliana*. PLoS Genet. 12:e1006263. 10.1371/journal.pgen.1006263, PMID: 27541584 PMC4991793

[ref148] Yamasaki H. Hayashi M. Fukazawa M. Kobayashi Y. Shikanai T. (2009). SQUAMOSA promoter binding protein–like7 is a central regulator for copper homeostasis in *Arabidopsis*. Plant Cell 21, 347–361. 10.1105/tpc.108.060137, PMID: 19122104 PMC2648088

[ref149] Yan J. Wang P. Wang B. Hsu C. C. Tang K. Zhang H.. (2017). The SnRK2 kinases modulate miRNA accumulation in *Arabidopsis*. PLoS Genet. 13:e1006753. 10.1371/journal.pgen.1006753, PMID: 28419088 PMC5413060

[ref150] Yang L. Xu M. Koo Y. He J. Poethig R. S. (2013). Sugar promotes vegetative phase change in *Arabidopsis thaliana* by repressing the expression of MIR156A and MIR156C. eLife 2:e00260. 10.7554/eLife.00260, PMID: 23538384 PMC3608266

[ref151] Yoine M. Ohto M. A. Onai K. Mita S. Nakamura K. (2006). The lba1 mutation of UPF1 RNA helicase involved in nonsense-mediated mRNA decay causes pleiotropic phenotypic changes and altered sugar signalling in *Arabidopsis*. Plant J. 47, 49–62. 10.1111/j.1365-313X.2006.02771.x, PMID: 16740149

[ref152] Yu S. Cao L. Zhou C. M. Zhang T. Q. Lian H. Sun Y.. (2013). Sugar is an endogenous cue for juvenile-to-adult phase transition in plants. eLife 2:e00269. 10.7554/eLife.00269, PMID: 23543845 PMC3610343

[ref153] Yu H. Cong L. Zhu Z. Wang C. Zou J. Tao C.. (2015). Identification of differentially expressed microRNA in the stems and leaves during sugar accumulation in sweet sorghum. Gene 571, 221–230. 10.1016/j.gene.2015.06.056, PMID: 26117170

[ref154] Zhang B. (2015). MicroRNA: a new target for improving plant tolerance to abiotic stress. J. Exp. Bot. 66, 1749–1761. 10.1093/jxb/erv013, PMID: 25697792 PMC4669559

[ref155] Zhang S. Liu Y. Yu B. (2014). PRL1, an RNA-binding protein, positively regulates the accumulation of miRNAs and siRNAs in *Arabidopsis*. PLoS Genet. 10:e1004841. 10.1371/journal.pgen.1004841, PMID: 25474114 PMC4256206

[ref156] Zhang H. Mao X. Jing R. (2011). SnRK2 acts within an intricate network that links sucrose metabolic and stress signaling in wheat. Plant Signal. Behav. 6, 652–654. 10.4161/psb.6.5.14945, PMID: 21448000 PMC3172830

[ref157] Zhao J. Missihoun T. D. Bartels D. (2017). The role of *Arabidopsis* aldehyde dehydrogenase genes in response to high temperature and stress combinations. J. Exp. Bot. 68, 4295–4308. 10.1093/jxb/erx194, PMID: 28922758 PMC5853279

[ref158] Zhao J. Missihoun T. D. Bartels D. (2018). The ATAF1 transcription factor is a key regulator of aldehyde dehydrogenase 7B4 (ALDH7B4) gene expression in *Arabidopsis thaliana*. Planta 248, 1017–1027. 10.1007/s00425-018-2955-1, PMID: 30027414

[ref159] Zheng J. Ma Y. Zhang M. Lyu M. Yuan Y. Wu B. (2019a). Expression pattern of FT/TFL1 and miR156-targeted SPL genes associated with developmental stages in *Dendrobium catenatum*. Int. J. Mol. Sci. 20:2725. 10.3390/ijms20112725, PMID: 31163611 PMC6600168

[ref160] Zheng Z. Xu X. Crosley R. A. Greenwalt S. A. Sun Y. Blakeslee B.. (2010). The protein kinase SnRk2.6 mediates the regulation of sucrose metabolism and plant growth in *Arabidopsis*. Plant Physiol. 153, 99–113. 10.1104/pp.109.150789, PMID: 20200070 PMC2862418

[ref161] Zheng C. Ye M. Sang M. Wu R. (2019b). A regulatory network for miR156-SPL module in *Arabidopsis thaliana*. Int. J. Mol. Sci. 20:6166. 10.3390/ijms20246166, PMID: 31817723 PMC6940959

